# Impact of heat and a rest-shade-hydration intervention program on productivity of piece-paid industrial agricultural workers at risk of chronic kidney disease of nontraditional origin

**DOI:** 10.1093/annweh/wxae007

**Published:** 2024-02-17

**Authors:** Erik Hansson, Kristina Jakobsson, Jason Glaser, Catharina Wesseling, Denis Chavarria, Rebekah A I Lucas, Heath Prince, David H Wegman

**Affiliations:** La Isla Network, 2219 California NW Unit 52, 20008 Washington, District of Columbia, United States; Occupational and Environmental Medicine, School of Public Health and Community Medicine, Sahlgrenska Academy, University of Gothenburg, Box 414, 413 90 Gothenburg, Sweden; La Isla Network, 2219 California NW Unit 52, 20008 Washington, District of Columbia, United States; Occupational and Environmental Medicine, School of Public Health and Community Medicine, Sahlgrenska Academy, University of Gothenburg, Box 414, 413 90 Gothenburg, Sweden; Occupational and Environmental Medicine, Sahlgrenska University Hospital, Box 414, 413 90 Gothenburg, Sweden; La Isla Network, 2219 California NW Unit 52, 20008 Washington, District of Columbia, United States; La Isla Network, 2219 California NW Unit 52, 20008 Washington, District of Columbia, United States; Unit of Occupational Medicine, Institute of Environmental Medicine, Karolinska Institute, Box 210, 171 77 Stockholm, Sweden; Occupational Health Management, Ingenio San Antonio/Nicaragua Sugar Estates Limited, Km. 119 Carretera León-Chinandega, Chichigalpa, Nicaragua; La Isla Network, 2219 California NW Unit 52, 20008 Washington, District of Columbia, United States; School of Sport, Exercise and Rehabilitation Sciences, University of Birmingham, Birmingham B15 2TT, United Kingdom; La Isla Network, 2219 California NW Unit 52, 20008 Washington, District of Columbia, United States; LBJ School of Public Affairs, University of Texas at Austin, 2315 Red River St, Austin, TX 78712,United States; La Isla Network, 2219 California NW Unit 52, 20008 Washington, District of Columbia, United States; University of Massachusetts Lowell, 1 University Avenue, Lowell, 01854 MA, United States

**Keywords:** chronic kidney disease, occupational medicine, heat stress, intervention, productivity

## Abstract

**Objectives:**

Assess the impact of environmental heat and a rest-shade-hydration (RSH) intervention against heat stress on productivity of piece-paid Mesoamerican sugarcane cutters. These workers are at a high risk of chronic kidney disease of non-traditional origin (CKDnt), from the severe heat stress they experience due to heavy work under hot conditions. RSH interventions in these populations improve kidney health outcomes, but their impact on productivity has yet to be examined.

**Methods:**

We accessed routine productivity data from seed (SC, *N* = 749) and burned (BCC, *N* = 535) sugarcane cutters observed over five harvest seasons with increasing RSH intervention at a large Nicaraguan sugarcane mill. Hourly field-site wet-bulb globe temperature (WBGT) was recorded by mill staff and summarized as a daily mean. Mixed linear regression was used to model daily productivity, adjusting for age (18–29, 30–44, and >45 years), sex, WBGT (<28, 28–29, 29–30, 30–31, and >31 °C) on the same and preceding day, harvest season (2017–18 to 2021–22), month, and acclimatization status (<1, 1–2, and >2 weeks).

**Results:**

There was an inverse dose–response relationship between SC productivity and WBGT on the same and preceding days, decreasing by approximately 3%/°C WBGT. Productivity increased during the study period, i.e. coinciding with RSH scale-up, by approximately 19% in SC and 9% in BCC.

**Conclusion:**

Agricultural worker productivity was expected lower on hotter days, strengthening the interest in all stakeholders to mitigate increasing global temperatures and their impact. Despite decreasing the total time allocated for work each day, an RSH intervention appears to result in increased productivity and no apparent loss in productivity.

**What’s Important About This Paper?**

Mesoamerican sugarcane workers have high heat exposure and related disease risk, but their economic vulnerability makes it a priority for these workers to maintain this employment. A clear effect of heat on productivity is displayed in a large study performed in real-world conditions. Workers in this study had increased productivity despite taking more rest, indicating that conscious efforts to reduce heat stress may lead to improved productivity that benefits both employers and employees.

## Introduction

Heat stress is a well-recognized occupational health hazard that increases physiological strain, heightens the risk of heat-related occupational injuries and illnesses, *and* decreases productivity and performance (Lucas, Epstein, and Kjellstrom 2014). Already, global warming is estimated to lead to a loss of 3.7 billion productive working hours in the agricultural sector in low- and middle-income countries (LMICs), a number predicted to increase (Levi, Kjellstrom, and Baldasseroni 2018; Romanello et al. 2022). Strengthening occupational heat stress prevention while minimizing productivity losses is, therefore, necessary to adapt to a warmer climate. There is accumulating evidence that rest-shade-hydration (RSH) workplace interventions prevent kidney injury in heat-stressed working populations at a high risk of chronic kidney disease of nontraditional origin (CKDnt) (Hansson et al. 2019; Glaser et al. 2020, 2022).

Workplace heat stress interventions may conceivably both decrease and increase worker productivity, i.e. worker production per time unit. With the implementation of regulated rest periods coupled with a fixed hour-to-end daily work, time available for productive work decreases as a larger proportion of the work shift is spent resting. Should this result in lower productivity it could lead to resistance from employers but also from piece-paid workers whose annual income is highly dependent on such work (Morera et al. 2020; Pacheco-Zenteno et al. 2021). However, a healthy, euthermic, and well-hydrated workforce may well be more productive during work periods. Studies have used empirical evidence from laboratory research and heat work–rest cycle regulatory guidelines to predict the impact of heat on physical work capacity (PWC), productivity, and macroeconomic outcomes (Foster et al. 2021; Zhao et al. 2021; Ioannou et al. 2022), but to our knowledge the number of studies reporting empirical findings on the association between heat and measured productivity in LMIC agricultural workers (e.g. Sahu, Sett, and Kjellstrom 2013; Sett and Sahu 2014; Dally et al. 2018; Sadiq, Hashim, and Osman 2019) is limited.

The Adelante Initiative is an ongoing multi-stakeholder RSH intervention that employs the PREP (Prevention Resilience Efficiency and Protection) research methodology, with the objective of reducing heat stress among sugarcane workers at Ingenio San Antonio (ISA), Chichigalpa, Nicaragua. We have published the apparent success of this RSH effort in reducing health impacts on the kidney (Hansson et al. 2019; Glaser et al. 2020, 2022). Based on the first and second harvests of the Adelante intervention, the company’s return on investment was estimated as a 22 cents gain in return per $1.00 invested in the RSH intervention (Prince 2020), but the impact on worker productivity over the first four intervention scale-up years remains to be quantified. In the present study, we utilize routine productivity data collected by the employer between 2017 and 2022 to assess the association between heat and individual-level productivity, and to understand how productivity has changed as an RSH intervention has been implemented and gradually improved.

## Methods

### Setting

Chichigalpa in Northwestern Nicaragua is characterized by a hot, tropical, lowland climate, and a vast sugarcane monoculture, managed by large sugar mills and their out-growers. Sugarcane cutting is predominantly seasonal, with most workers employed during the November–April harvest season, coinciding with the dry season. Harvesting is conducted using mechanical harvesters, or by manual workers cutting the sugarcane using a machete. Manual sugarcane harvest workers are piece-paid, and the income from harvest work represents a substantial proportion of their yearly earnings.

Sugarcane can be harvested either for producing sugar, biofuels, bioplastics, etc., or for making seedlings to be sown in new or previously cultivated sugarcane fields. Sugarcane fields are typically burned prior to manual harvesting for cane to be further processed while cutting of seedlings uses living, green cane. Burned sugarcane is harvested from November to April, and green sugarcane is cut from November to July. At ISA, manual cutting of burned and green sugarcane is performed by different groups of workers, burned cane, and seed cutters (henceforth BCC and SC). BCC are all male, while women make up ~20% of the SC group at ISA. Both groups of workers cut the sugarcane just above ground level using a machete and remove the tops. Following this, the SC then cut the sugarcane into ~30 cm pieces and made bundles of approximately 30 pieces, while BCC stacked the whole sugarcane in piles. Work is organized into groups (*cuadrillas*) each including approximately 60 workers who arrive at work by the same bus and have a common supervisor and health promotor (Pacheco-Zenteno et al. 2021).

This study addresses the impact of the heat stress prevention program over the five harvests (labeled H1 to H5) that occurred from 2017 to 2022. Existing workplace conditions and heat stress prevention measures were observed during H1, and recommendations for enhanced RSH heat stress prevention were then provided between H2 and H5. Importantly, it was clarified that breaks were mandatory and should be seen as a regular part of the work tasks. This became accepted and enforced by work supervisors, who came to see it as their job to oversee breaks on the piece-paid cutters (Pacheco-Zenteno et al. 2021). During the study period, the workday ended at 2 pm for SC and at noon for BCC. The proportion of rest under shaded tents before noon for SC increased from one 20 min break in H1 (i.e. 5% of the workday before noon was spent resting) to hourly 10 min breaks in H2 (11%), to 10–20 min of hourly rest in H3 and the following harvests (22%) (Glaser et al. 2022). The SC had a 30 min lunch break at noon and then continued without breaks until 2 pm in H1–H2, and thereafter had a 20 min break at 1 pm. For BCC, the proportion of rest before noon was increased from 14% to 17% between H1 and H2, and then to 22% in H3 (Glaser et al. 2022). Between H1 and H2, shade tents were improved by changing to a fabric providing slightly more cooling (Glaser et al. 2020), and the movement of tents closer to the workers was stressed, as was the importance of hydration, including rehydration solution. Both water and rehydration solution were provided by the employer, and freely available in the shade tents. Workers were not forced or incentivized to drink, but hydration practices were encouraged and supervised by the health promotors. An existing acclimatization program over the first 2 weeks of the harvest, in which workers gradually had increasing workday length and production goals, was in place during all years.

During the covid-19 pandemic, sugarcane harvesting continued as usual; workers however wore face masks, washed their hands before and after work shifts, were requested to keep physical distance, were monitored for signs and symptoms of covid-19, and tested by mill hospital staff if needed.

### Data collection

Daily productivity data were available at an individual level only for SC. For these workers, the day-by-day individual production was obtained from company records of the SC supervisor counts of the numbers of seed sugarcane bundles/cutters for each day for each individual for all harvests from H1 through H5. For BCC, however, the equivalent individual daily productivity data were not available. The BCC was self-organized into subgroups of approximately 10 individuals who shared a common daily payment based on the subgroup production. These subgroups could change on a daily or weekly basis. Company records for individual BCC were available for the average daily productivity over the entire harvest for H1 and H2. The company was able to provide data for H3–H5 that recorded the number of tons of burned sugarcane cut by each subgroup of workers each day, the worker group membership, and whether each individual worked on any given day.

The average daily wet-bulb globe temperature (WBGT) was obtained from field-site measurements at ~1 m from the ground using a QuesTemp (H1 and 2) or Kestrel (H2–5) device. QuesTemp measurements were entered manually each hour, while the Kestrel device logged data every 30 min. Measurements were taken among outdoor workers performing a variety of outdoor jobs for the mill, but always within ~10 km from and always at a similar altitude as the workplaces included in the current study. The average WBGT between 8 am and 2 pm each day was calculated and categorized into <28, 28–29, 29–30, 30–31, and >31 °C.

The number of days into harvest for each individual was calculated and categorized to 0–6, 7–13, and >13 days to allow accounting for the acclimatization program at ISA.

### Statistical analysis

Stata version 17 was used for all statistical analyses.

#### Burned cane cutters (BCC).

Three outliers recorded to have been cutting on average >12 tons/day (observed for 1 day only) and <2 tons/day (unclear observation durations) were removed. The distribution of average tons of burned sugarcane harvested/day/harvest among the remaining BCC followed approximately normal distributions with similar variances for each harvest (Supplementary Fig. S1). Individual daily average tons of burned sugarcane cut were compared for each harvest, using *t*-tests comparing the first harvest with each of the remaining. Both unpaired and paired *t*-tests were performed as some workers worked multiple harvests.

#### Seed cutters (SC).

Days without same day WBGT measurements were excluded, as were non-Mondays without WBGT measurements from the preceding day. Outliers in productivity data which were so extreme that they were deemed more likely due to data entry error than true were removed (<30 and >400 bundles/day, *N* = 366 out of 101 028). The number of bundles produced per day was transformed into the natural log as this distribution was closer to a normal distribution. Ten-digit preference was detected as one possible violation of assumptions of normal distribution (Supplementary Fig. S2A). Specifically, 100 bundles were disproportionately often recorded. This specific digit preference may both be due to workers choosing to aim for such an even number for their daily production, and supervisors rounding the recorded number of bundles. This phenomenon occurred to a similar extent across harvest years and environmental heat (Supplementary Fig. S2B and C), and it will thereby likely lead to a reduction in the possibility for a statistical analysis to detect an effect, rather than spuriously introduce one.

The log-transformed number of seed bundles produced each day was estimated using mixed linear regression models employing the *xtreg* command, fitted using maximum likelihood estimation. Models included a random intercept for workers and fixed effects for harvest year, sex, and WBGT category on the same and preceding day (Sundays were categorized separately), i.e. a 1-day distributed lag model. This regression model assumes that individual-level residuals (*µ*) and residuals within individuals (*ε*) are normally distributed, homoscedastic, and independent. This was evaluated by plotting the residuals in a histogram and in a scatter plot versus the predicted values. As a sensitivity analysis, we evaluated models allowing for heteroscedasticity (by specifying the vce(robust) option, Supplementary Table S1). Correlated *ε* within individuals, varying with time, is another plausible violation of model assumptions in this context, as productivity on adjacent days is likely correlated. Therefore, as a sensitivity analysis, we evaluated models with autocorrelation between adjacent days within individuals using the *xtregar* command (Supplementary Table S1).

Separate models were run for men and women to understand the influence of biological sex on heat-related changes in productivity.

## Results

### Burned cane cutters (BCC)

A total of 533 BCC observed over 1094 worker-harvests were included (Table 1). Due to the incomplete data on productivity for BCC, only the overall daily averages for each harvest were examined. The harvest-average daily number of tons cut fluctuated around 6 tons/day per worker, however generally approximately 9% higher in H3–5 than in H1–2 (Table 1).

**Table 1.** Average burned cane cutter production at Ingenio San Antonio, Chichigalpa, Nicaragua during five harvest periods.

Harvest	Burned sugarcane tons cut/day/worker		Worker-harvests		*t*-test							
				Unpaired				Paired				
	Mean	SD		Mean difference	95% CI		p	Paired observations	Mean difference	95% CI		*P*
					Low	High				Low	High	
1. 2017–18	5.7	1.0	381	Reference				Reference				
2. 2018–19	5.6	1.0	193	−0.08	−0.25	0.09	0.35	131	−0.03	−0.12	0.07	0.56
3. 2019–20	6.3	0.8	180	0.55	0.38	0.71	<0.001	108	0.40	0.28	0.52	<0.001
4. 2020–21	5.9	0.8	173	0.23	0.06	0.40	0.007	93	0.12	0.00	0.24	0.04
5. 2021–22	6.2	0.8	167	0.48	0.31	0.65	<0.001	87	0.32	0.16	0.47	<0.001

### Seed cutters

A total of 716 individual SC workers with a total of 100 662 worker-days over five harvests were included (Table 2). For this job category, the data were sufficient to examine daily productivity differences across the harvest. Eighty percent of the workdays were performed by male workers. Mean seed cutter bundle productivity was higher among males, in workers younger than 45 years, and in the later parts of harvest than the first 2 weeks when shorter work periods occur during acclimatization. Productivity increased during each year of the study period compared to the previous year, so that workers in H5 produced approximately 19% more than in H1. The variance of random effects at the individual level, *µ*, was 19% (standard error 0.56), and the variance of residuals within the individual, *ε*, was 35% (standard error 0.07).

**Table 2.** Determinants of daily seed sugarcane bundles cut per worker at Ingenio San Antonio, Chichigalpa, Nicaragua during five harvest periods.

	Seed sugarcane bundles produced		Worker-days	Regression model			
	Mean	SD		Main estimate (% additional bundles)	95% CI		*P*
					Low	High	
Harvest							
1. 2017–18	129	49	15,632	Reference			
2. 2018–19	130	42	21,469	5.8	5.0	6.6	<0.001
3. 2019–20	135	49	21,528	8.2	7.3	9.1	<0.001
4. 2020–21	146	52	19,675	12.2	11.3	13.2	<0.001
5. 2021–22	149	53	22,358	19.2	18.2	20.2	<0.001
WBGT category, same day							
<28	140	54	6218	Reference			
28–29	139	50	16,370	−2.5	−3.4	−1.7	<0.001
29–30	142	50	35,031	−2.7	−3.6	−1.9	<0.001
30–31	137	48	25,995	−5.0	−5.8	−4.1	<0.001
31+	130	50	17,048	−8.3	−9.2	−7.4	<0.001
WBGT category, previous day							
Sunday	141	53	16,604	−1.1	−2.1	0.0	0.42
<28	139	52	4438	Reference			
28–29	142	50	14,404	0.1	−1.0	1.1	0.91
29–30	140	49	29,908	−2.8	−3.8	−1.8	<0.001
30–31	136	48	20,692	−4.2	−5.2	−3.2	<0.001
>31	130	50	14,616	−6.8	−7.9	−5.7	<0.001
Sex							
Men	143	51	80,195	Reference			
Women	121	39	20,467	−13.4	−16.5	−10.2	<0.001
Days into harvest (acclimatization period)							
0–6	91	34	1928	−24.5	−25.6	−23.4	<0.001
7–13	121	39	2124	−1.9	−3.3	−0.6	0.005
>13	140	50	96,610	Reference			
Month							
November	77	34	770	−43.6	−44.8	−42.3	<0.001
December	123	41	16,485	−12.0	−12.5	−11.4	<0.001
January	146	50	22,409	Reference			
February	145	52	21,053	−1.1	−1.7	−0.6	<0.001
March	138	48	22,563	−5.3	−5.8	−4.8	<0.001
April	138	51	15,173	−1.7	−2.4	−1.0	<0.001
May	134	55	2209	−4.8	−6.2	−-3.4	<0.001
Age							
18–29	140	51	62,012	Reference			
30–44	136	48	35,995	−0.7	−1.8	0.3	0.18
>45	120	36	2655	−5.8	−9.3	−2.3	0.001
Intercept				130.6	128.0	133.2	

**Fig. 1.**
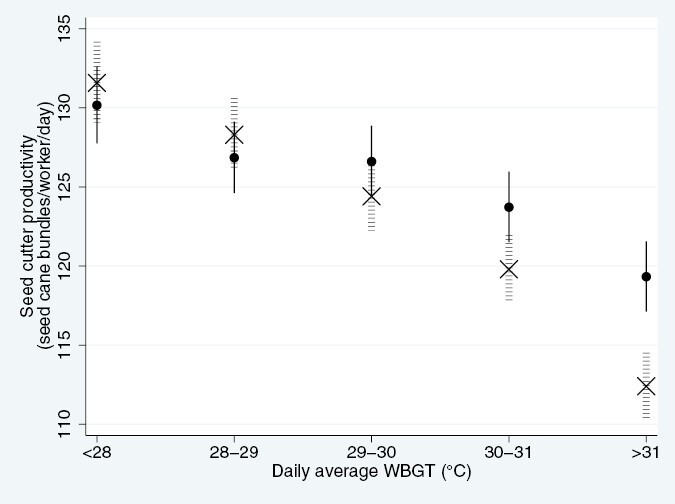
Association between external heat and seed cutter productivity, accounting for sex, age, month, acclimatization and harvest. The *x*-axis display modeled estimates in an 18–30 year old male worker, in the baseline year (January, 2017–2018), having worked more than 14 days (thus acclimatized). The solid black dots [●] and lines describe the heat-productivity association on a Monday, i.e. after a rest day, and the crosses [X] and horizontal dashed lines describe the heat-work association on a day preceded by a non-Monday in the same WBGT category, i.e. the accumulated effect of 2 days of equal heat exposure.

We sought to investigate how the same-day (lag day 0) and preceding day (lag day 1) environmental heat affected productivity. Higher WBGT was associated with a reduced productivity on lag days 0 and 1, with an approximate 3% loss of productivity per each degree WBGT above 28 °C when summarizing the effect of both days (Fig. 1). Mondays, i.e. the day after a rest day, had a similar productivity as days preceded by workdays with WBGT <29 °C. However, temperatures >29 °C on the preceding workday led to a reduction in productivity.

**Fig. 2.**
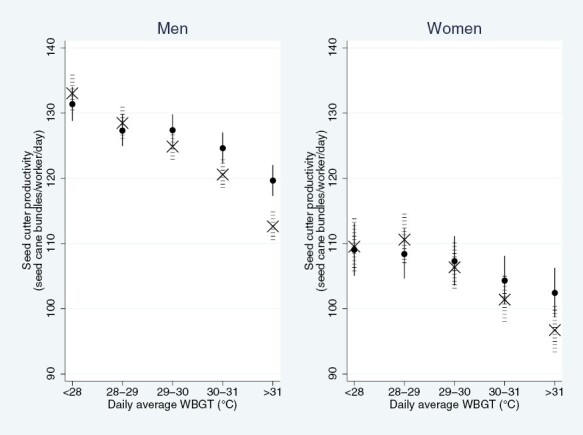
Association between environmental heat stress (WBGT) and seed cutter productivity, stratified by sex, accounting for age, month, acclimatization and harvest. The *x*-axis display modeled estimates in an 18–30 year old seed cutter, in the baseline year (January, 2017–2018), having worked more than 14 days (thus acclimatized). The solid black dots [●] and lines describe the heat-productivity association on a Monday, i.e. after a rest day, and the crosses [X] and horizontal dash lines describe the heat-work association on a day preceded by a non-Monday in the same WBGT category, i.e. the accumulated effect of 2 days of equal heat exposure.

The difference between male and female productivity was narrower at the higher WBGT levels, meaning there was a smaller reduction in productivity among female than male workers when the WBGT increased (Fig. 2).

Histograms of the residuals *µ* and *ε* show that these were normally distributed (Supplementary Fig. S3). Visual inspection of the residual plots for *µ* and *ε* versus predicted values suggested the presence of heteroscedasticity (Supplementary Fig. S4). Although accounting for heteroscedasticity increased standard errors, and accounting for autocorrelation led to somewhat reduced effect sizes, the above findings overall remained (Supplementary Table S1).

## Discussion

Environmental heat stress had an expected negative effect on seed cutter productivity, with a magnitude of approximately 3% decrease/°C WBGT when heat stress increased from <28 to >31 °C WBGT. This is comparable to a 5% and 2% decrease/°C WBGT previously reported from Indian rice harvest and brick workers respectively (Sahu, Sett, and Kjellstrom 2013; Sett and Sahu 2014). A recent empirical model derived from laboratory studies on the impact of heat on PWC, i.e. the maximum physical work output that can be reasonably expected from an individual performing moderate to heavy work over an entire shift, suggested an 18% PWC reduction between 28 and 31 °C WBGT (Foster et al. 2021), not unlike the 15% reduction when going from a <28 °C day preceded by a rest day to a >31 °C day preceded by an equally hot day seen in this study. Interestingly, the heat-productivity association remained with a lag of one day, indicating that workers did not completely recover the day after working heavily in heat. This is in line with heat morbidity and mortality effects, where increases in hospitalizations from kidney disease and deaths from coronary heart disease are elevated at a lag of 0–2 days (Hu et al. 2021; Wen et al. 2022).

Heat-related productivity reductions affected workers throughout most of the harvest season, with the reference category of <28 °C only making up approximately 6% of the worked days. Further, we cannot rule out heat-related productivity occurring in the reference heat category of <28 °C as 27 °C WBGT is considered to represent substantial heat stress at which declining PWC would be expected (Foster et al. 2021).

Productivity consistently increased in SC during the 5-year study period. Among the BCC, productivity in H3–5 was generally 9% higher than in H1–2, despite a slight dip during H4 coinciding with the covid-19 peak in this population. Records from ISA in 2012–2015 indicate BCC cut on average approximately 6 tons/day, despite working 8 h/day rather than 4.67 h/day as from H3 onwards (Supplementary Fig. S5). The RSH intervention scale-up stipulated that a larger proportion of the workday must be spent resting, however, the actual change in the proportion of rest each day for BCC was relatively less (14–22%) than for SC (10–25%). It should be acknowledged that in addition to the RSH intervention, the mill has adapted SC work practices during the study period so that these workers now cut sugarcane at an ideal stage of maturity (after 6–7 months of growth) and predominantly at good soil fields where the sugarcane typically grows more up-right, factors which likely contributed to improved productivity rates in this group. Regardless, the RSH intervention, which significantly reduced the number of hours worked per day, did not negatively impact productivity. Further, effective heat stress interventions should include optimizing the workers’ tasks to minimize unnecessary and strenuous activities.

It appears that the RSH intervention improved workers’ productivity during active work periods. Preventing hyperthermia (Lucas et al. 2023) and dehydration (Glaser et al. 2020) via regulated hourly shaded rest periods with easy access to hydration beverages could have allowed workers to increase their productivity, just as endurance sports athletes perform better when hyperthermia (Jones et al. 2012) and dehydration (Cheuvront, Carter, and Sawka 2003) are prevented. Fewer workers developing fever or other symptoms, or biochemical signs of systemic inflammation, were observed with the RSH intervention (Glaser et al. 2020, 2022; Hansson et al. 2020), likely also contributing to maintaining productivity.

The higher productivity in workers aged <45 years as compared to females and older males is likely to be largely a consequence of population-level differences in cardiorespiratory capacity and strength. In a small field-based observational study we have previously shown that women seed cutters work at a higher relative work intensity (based on %HR_max_) than their male counterparts (Lucas et al. 2023). However, in the current study women workers had a lower sensitivity to heat-related productivity loss (smaller % productivity decline from <28 to >31 WBGT°C (Fig. 2)). It may be that women workers adopt better pacing strategies than young male workers. In nonelite marathon runners, compared with men, women show a pacing advantage, which further increases with warmer environmental conditions (Trubee et al. 2014). Other behavioral factors (e.g. being the primary or sole income earner) may also contribute to the higher productivity seen in younger male workers. Consistent with the acclimatization program implemented at ISA and the expected effects of physiological acclimatization to work in heat, there was an increase in productivity from the second week of the harvest onwards.

### Limitations

Measurement error can contribute to estimating study variables successfully. The productivity loss seen here may be underestimated due to measurement error arising from WBGT and productivity being aggregated over a whole working day rather than hour-by-hour. Another source of measurement error is that heat assessments were not always performed in the workers’ immediate vicinity but could be in a nearby field. However, the WBGT recordings across different locations at ISA were consistent on the same day in this low-land plantation area.

We did not explore longer lags than 1 day due to the complexities of considering the effects of the rest day on Sundays over longer lag periods. It is conceivable that more long-lasting or cumulative heat effects contribute to fatigue, ill health, and reduced productivity over several workdays, the working week, or harvest season, and that such effects further reduce productivity in addition to the short-term effects seen here. An example of such a longer-term effect of heat stress on productivity is when workers fall ill with asymptomatic or symptomatic kidney injury, in many cases necessitating sick leave, which occurred to a high degree at ISA prior to the RSH intervention, as previously reported (Glaser et al. 2020, 2022). Early evidence exists that the RSH intervention might translate into improved long-term kidney health prognosis (Glaser et al. 2022), retaining workers in the workforce who would otherwise fall ill and possibly die. Productivity losses from such labor supply loss and absenteeism are not considered in this study, which focused solely on presenteeism, i.e. productivity losses among workers present at the workplace (Zhao et al. 2021). Estimating the total productivity losses due to heat is complex as these losses occur on many different levels (Zhao et al. 2021).

## Conclusion

As the climate gets warmer, reduced agricultural worker output from increasing workplace heat stress, as described in this study, will reduce agricultural productivity in addition to other climate change-related factors such as extreme weather events and drought. However, rather than reducing productivity, implementation of an RSH intervention benefitted workers’ health and productivity while providing a positive return on investment for the employer. Conscious and active efforts on many levels are required to mitigate increasing global temperatures and implement suitable worker protections and practices effectively.

## Supplementary Material

wxae007_suppl_Supplementary_Material

## Data Availability

The data underlying this article will be shared on reasonable request to the corresponding author.
