# Supplementary material for: Impact of heat and a rest-shade-hydration intervention program on productivity of piece-paid industrial agricultural workers at risk of chronic kidney disease of nontraditional origin
Source: Ann Work Expo Health. 2024 Feb 17;68(4):366–75. doi: 10.1093/annweh/wxae007 (PMC11033565; doi:10.1093/annweh/wxae007)
Supplement: wxae007_suppl_Supplementary_Material [file wxae007_suppl_supplementary_material.pdf]

Impact of heat and a rest-shade-hydration intervention program on productivity of piece-paid industrial agricultural workers at risk of chronic kidney disease of non-traditional origin

Erik Hansson [1, 2], Kristina Jakobsson [1, 2, 3], Jason Glaser [1], Catharina Wesseling [1 4], Denis Chavarría [5], Rebekah A. I. Lucas [1, 6], Heath Prince [1, 7] David H. Wegman [1, 8]

[1] La Isla Network, Washington, District of Columbia, USA

[2] School of Public Health, University of Gothenburg Sahlgrenska Academy, Gothenburg, Sweden

[3] Occupational and Environmental Medicine, Sahlgrenska University Hospital, Gothenburg, Sweden

[4] Institute of Environmental Medicine, Unit of Occupational Medicine, Karolinska Institute, Stockholm, Sweden

[5] Occupational Health Management, Ingenio San Antonio/Nicaragua Sugar Estates Limited, Chichigalpa, Nicaragua

[6] School of Sport, Exercise and Rehabilitation Sciences, University of Birmingham, Birmingham, UK

[7] LBJ School of Public Affairs, University of Texas at Austin

[8] University of Massachusetts Lowell, Lowell, Massachusetts, USA

Supplement figures and tables

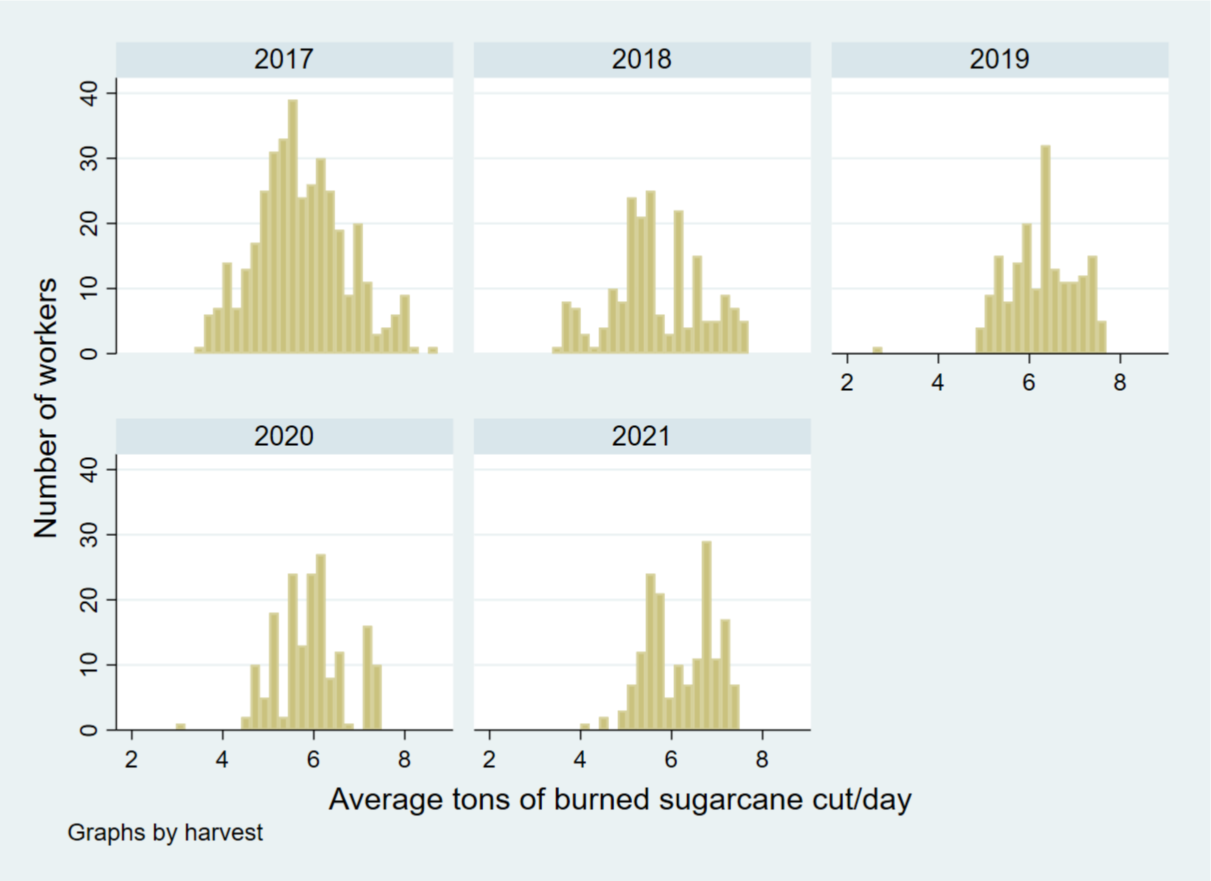

Supplement figure 1: Average tons of burned sugarcane cut per day, for each of the harvests 2017-2018, to 2021-2022.

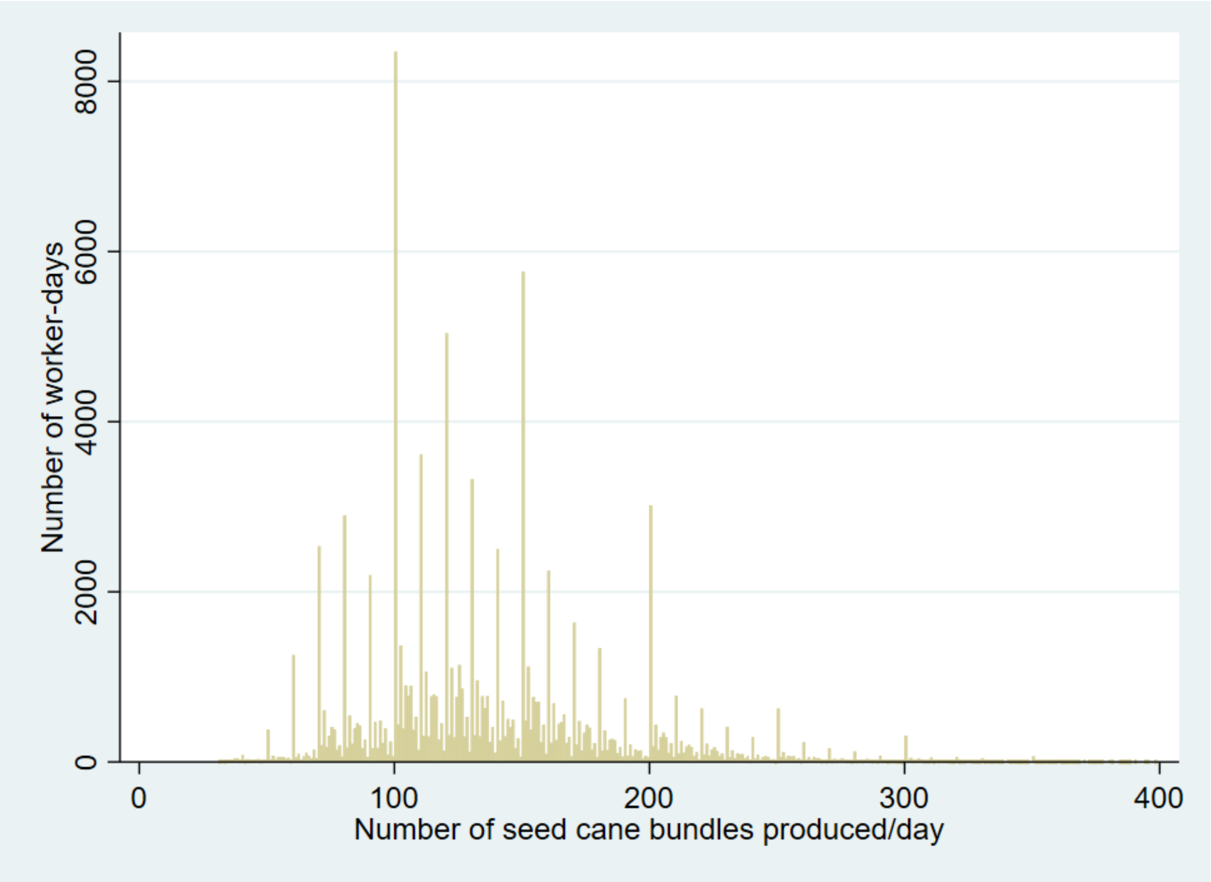

Supplement Figure 2A: Number of seed sugarcane bundles produced per day. The preference towards producing or recording numbers ending with zero can be seen.

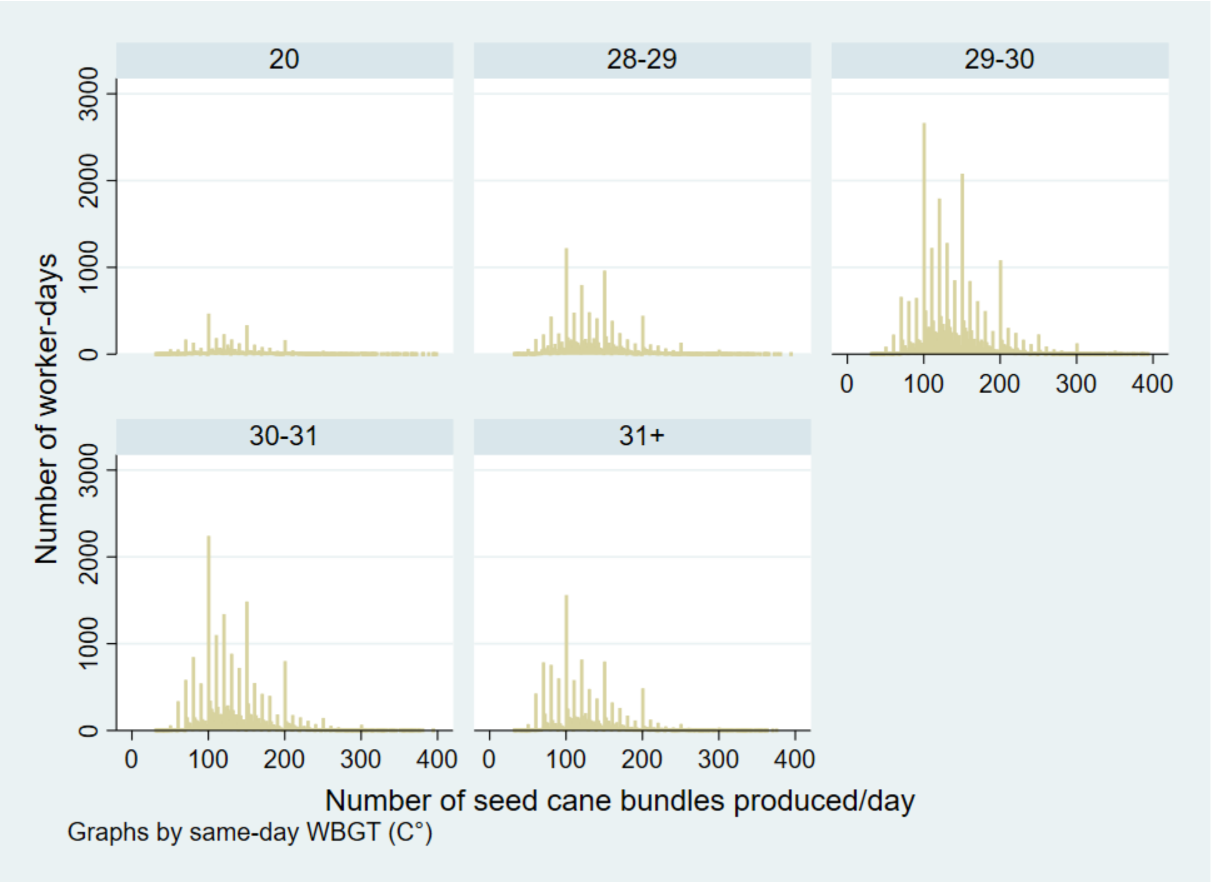

Supplement Figure 2B: Number of seed sugarcane bundles produced per day, by same-day heat

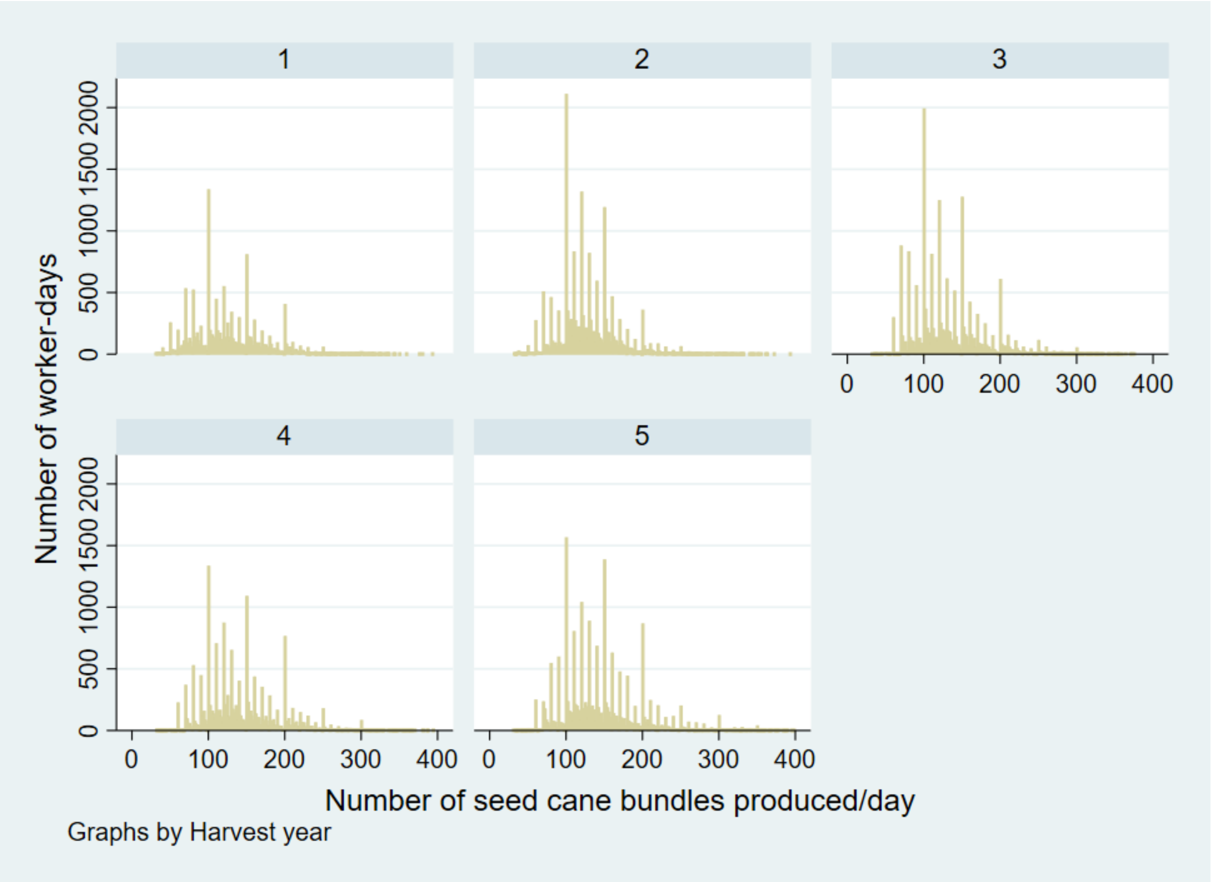

Supplement Figure 2C: Number of seed sugarcane bundles produced per day, by harvest

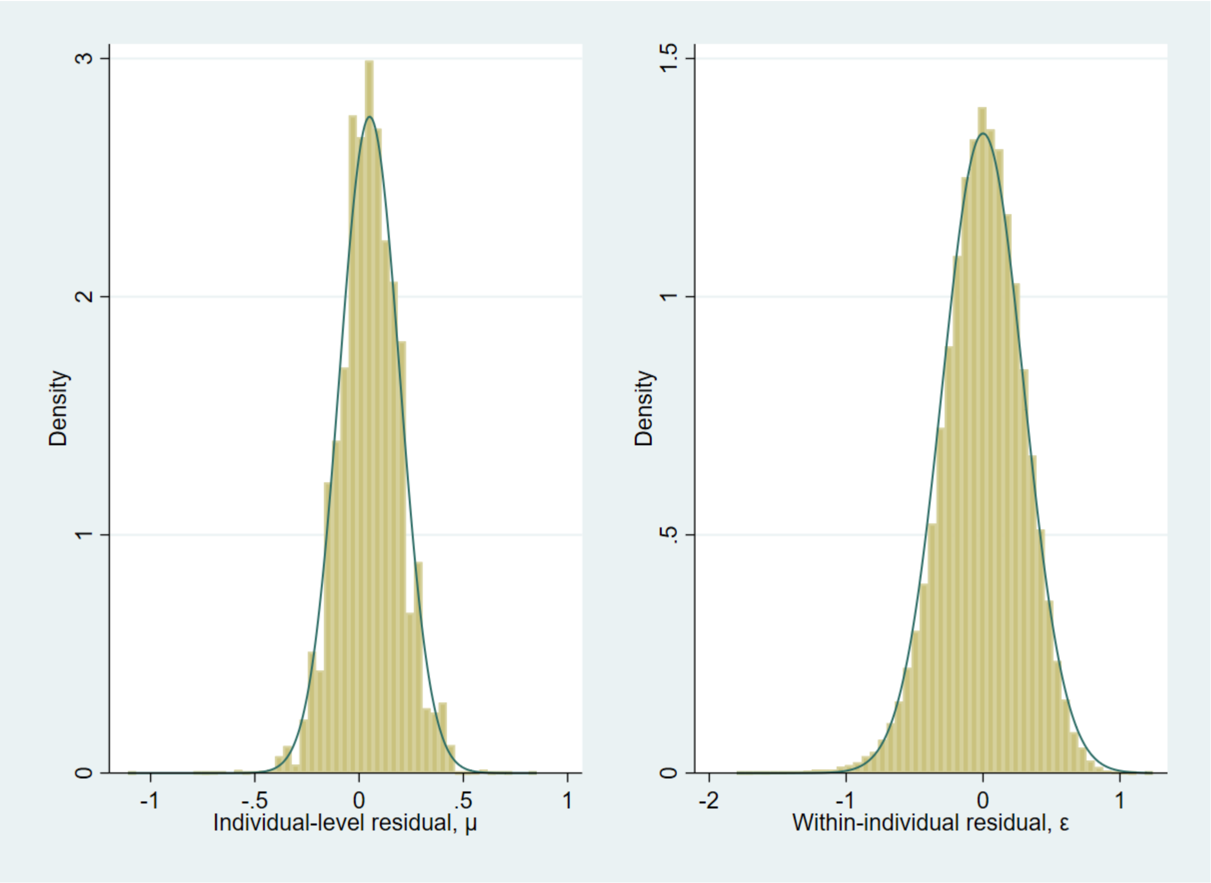

Supplement figure 3. Distribution of residuals for the seed sugarcane bundle productivity at individual and within-individual level.

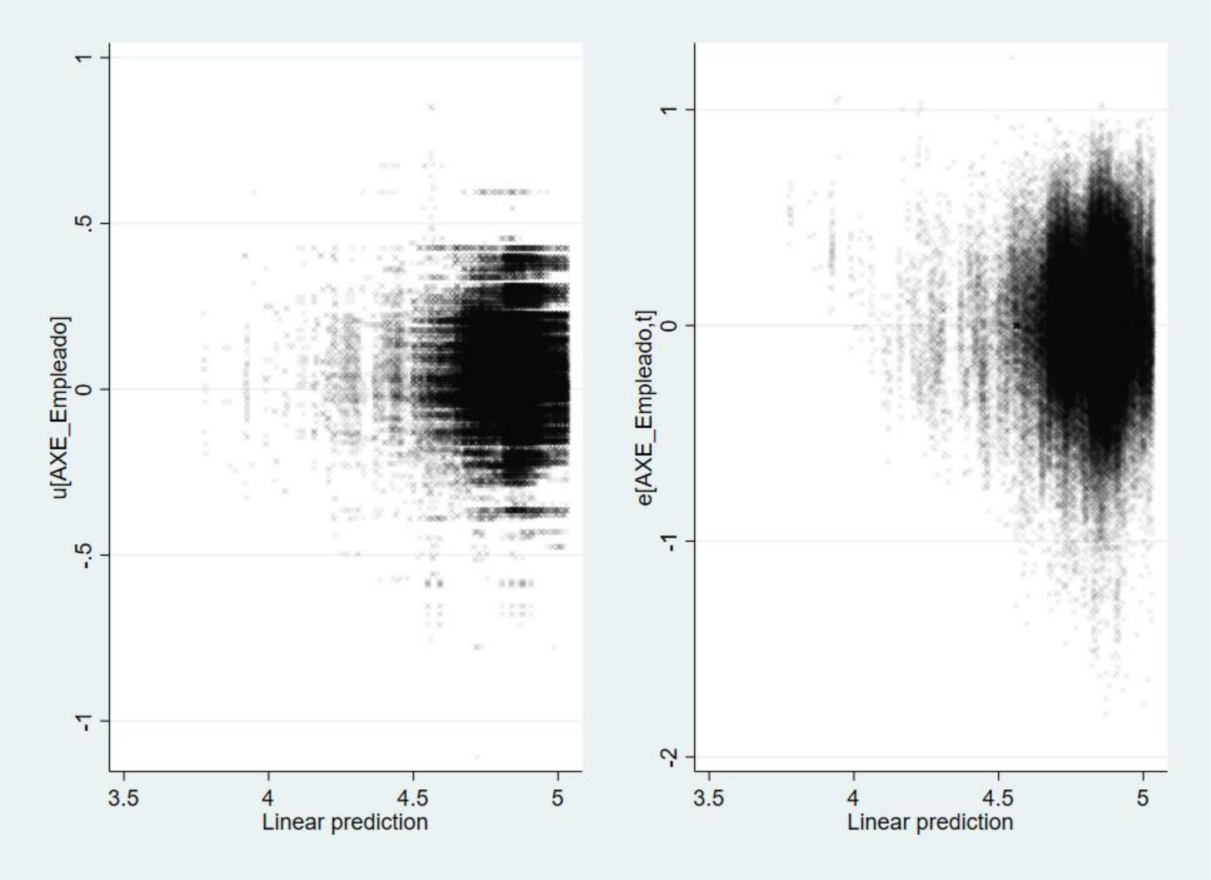

Supplement figure 4: Association between residuals for the seed sugarcane bundle productivity at individual and within-individual level and model predicted seed sugarcane productivity.

Supplement Table 1: Regression model estimates for main reported model and models allowing for violations of specific model assumptions.

	Main model	Allowing for heteroscedasticity	Allowing for autocorrelation within individuals
Harvest year			
1. 2017-18	Reference	Reference	Reference
2. 2018-19	6 (5,7)	6 (4,7)	5 (4,6)
3. 2019-20	8 (7,9)	8 (6,10)	7 (6,8)
4. 2020-21	12 (11,13)	12 (10,14)	11 (10,13)
5. 2021-22	19 (18,20)	19 (17,22)	17 (16,19)
Heat in the preceding day (WBGT)			
Sunday	-1 (-2,0)	-1 (-2,0)	0 (-1,1)
<28	Reference	Reference	Reference
28-29	0 (-1,1)	0 (-1.1)	0 (-1,1)
29-30	-3 (-4,-2)	-3 (-4,-2)	-2 (-3,-1)
30-31	-4 (-5,-3)	-4 (-5,-3)	-3 (-4,-2)
31+	-7 (-8,-6)	-7 (-8,-6)	-5 (-6,-4)
Same-day heat (WBGT)			
<28	Reference	Reference	Reference
28-29	-3 (-3,-2)	-3 (-4,-1)	-2 (-3,-1)
29-30	-3 (-4,-2)	-3 (-4,-2)	-1 (-2,-1)
30-31	-5 (-6,-4)	-5 (-6,-4)	-3 (-4,-2)
31+	-8 (-9,-7)	-8 (-9,-7)	-7 (-8,-6)
Sex			
Men	Reference	Reference	Reference
Women	-13 (-17,-10)	-13 (-16,-10)	-13 (-16,-10)
Time worked previously in the same harvest (days)			
0-6	-25 (-26,-23)	-25 (-26,-23)	-23 (-24,-21)
7-13	-2 (-3,-1)	-2 (-3-0)	-3 (-5,-1)
>13	Reference	Reference	Reference
Month			
November	-44 (-45,-42)	-44 (-46,-41)	-41 (-43,-40)
December	-12 (-13,-11)	-12 (-13,-11)	-13 (-14,-12)
January	Reference	Reference	Reference
February	-1 (-2,-1)	-1 (-2,0)	-1 (-2-0)
March	-5 (-6,-5)	-5 (-6,-5)	-5 (-6,-4)
April	-2 (-2,-1)	-2 (-3,-1)	-2 (-3,-1)
May	-5 (-6,-3)	-5 (-7,-3)	-6 (-8,-4)
Age (years)			
18-29	Reference	Reference	Reference
30-44	-1 (-2,0)	-1 (-3,1)	0 (-2,1)
>45	-6 (-9,-2)	-6 (-12,0)	-7 (-12,-2)
Intercept	131 (128,133)	131 (127,134)	130 (127,132)
Sigma μ	19 (18,20)	19 (18,21)	15
Sigma ε	35 (35,35)	35 (34,35)	33

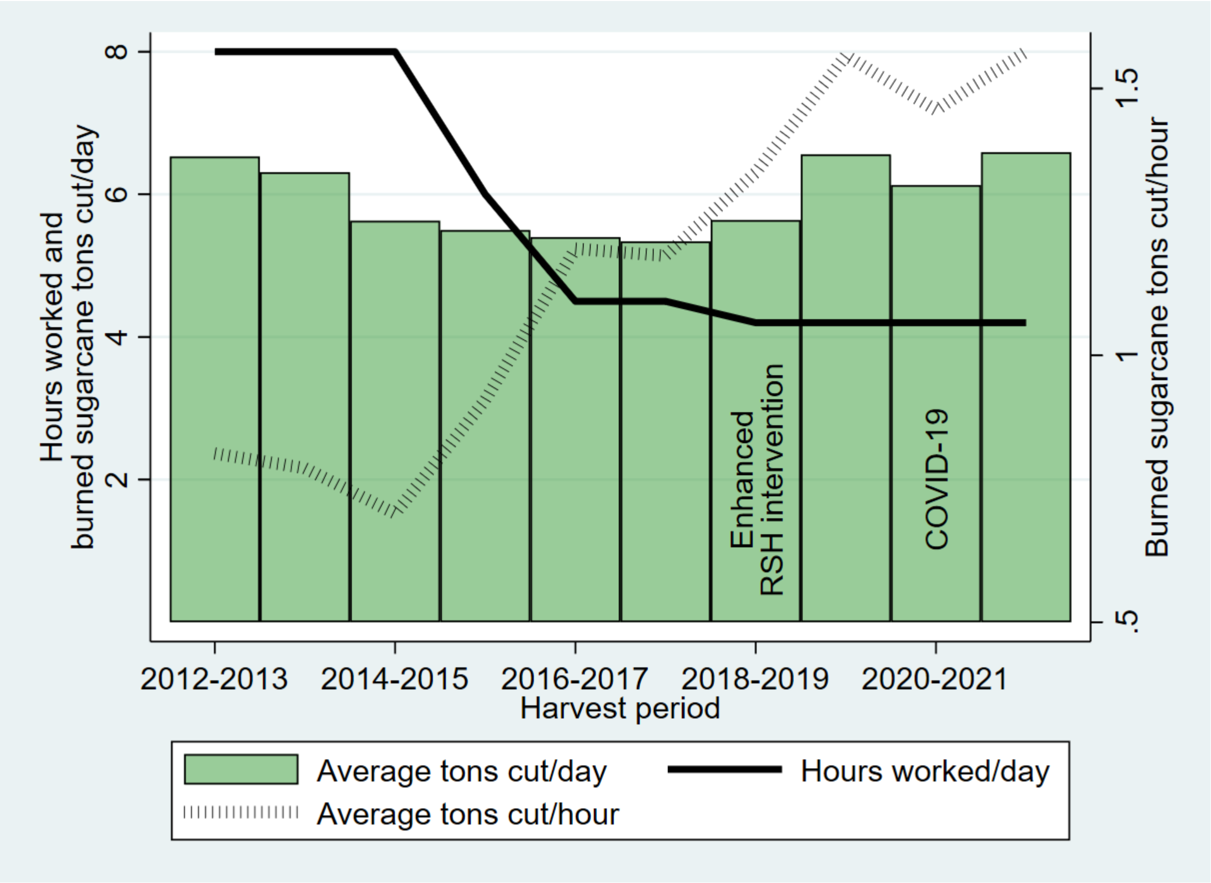

Supplement figure 5. Hours worked and average tons of burned sugarcane cut by ISA workers from 2012-2022.
